# Endophytic Bacteria Potentially Promote Plant Growth by Synthesizing Different Metabolites and their Phenotypic/Physiological Profiles in the Biolog GEN III MicroPlate^TM^ Test

**DOI:** 10.3390/ijms20215283

**Published:** 2019-10-24

**Authors:** Małgorzata Woźniak, Anna Gałązka, Renata Tyśkiewicz, Jolanta Jaroszuk-Ściseł

**Affiliations:** 1 Department of Agricultural Microbiology, Institute of Soil Science and Plant Cultivation—State Research Institute, 24-100 Puławy, Poland; agalazka@iung.pulawy.pl; 2 Department of Industrial and Environmental Microbiology, Faculty of Biology and Biotechnology, Maria Curie-Skłodowska University, 20-033 Lublin, Poland; renata.tyskiewicz@poczta.umcs.lublin.pl (R.T.); jolanta.jaroszuk-scisel@poczta.umcs.lublin.pl (J.J.-Ś.); 3 Military Institute of Hygiene and Epidemiology, Lubelska 2, 24-100 Puławy, Poland

**Keywords:** bacterial endophytes, physiological profiles, plant growth promoting properties

## Abstract

Endophytic bacteria, as the most promising components of effective, biofertilizers biostimulating and biocontrol preparations, should be very intensively obtained from various plants and studied in terms of the conditions determining the potential ability to promote plant growth. For this reason, endophytic bacteria have been isolated from both stems and roots of up to six systematically distant species of vascular plants: one species belonging to the seedless vascular plants (Monilophyta), and five seed plants (Spermatophyta). The 23 isolated strains represented nine genera: *Delftia*, *Stenotrophomonas*, *Rhizobium*, *Brevundimonas*, *Variovorax*, *Achromobacter*, *Novosphingobium*, *Comamonas* and *Collimonas*, notably which were closely related—belonging to the phylum Proteobacteria. *Stenotrophomonas* sp. strains showed the greatest ability to synthesize indole-3-acetic acid (IAA)-like compounds, while *Achromobacter* sp. strains produced the highest levels of siderophores. The presence of the *nifH* gene and nitrogen binding activity was demonstrated for 95% of the strains tested. *Stenotrophomonas maltophila* (ES2 strain) showed the highest metabolic activity based on Biolog GEN III test. The ability to solubilize phosphate was determined only for three tested strains from genus: *Delftia*, *Rhizobium* and *Novosphingobium*. The presented work demonstrated that the metabolic and phenotypic properties of plant growth-promoting endophytes are correlated with the genus of bacteria and are not correlated with the host plant species or part of plant (stem, root).

## 1. Introduction

At present, agricultural production depends on the large-scale use of chemical fertilizers. As such, these fertilizers have become the basic elements of modern agriculture, as they provide essential nutrients for plants, such as nitrogen, phosphorus and potassium. However, excessive use of fertilizer can cause unpredictable environmental effects, and have a negative impact on the physical, chemical and biological properties of soil. Contamination of groundwater, eutrophication of reservoirs, loss of soil fertility, a reduction in biodiversity, accumulation of harmful compounds in soils, concomitant acid rain and detrimental effects on the ozone layer are just some of the negative outcomes of using chemical fertilizers, indicating a need to find suitable alternatives [1,2]. Moreover, based on data from the Food and Agriculture Organization (FAO), the global population for 2025 is estimated to be almost 8.5 × 10^9^ people. Population growth will require an improvement in agricultural productivity [3,4]. The expected world population increase, increasing demand for the production of healthy food, reduced arable land through urban development, climate change, as well as the increasing environmental degradation therefore intensive fertilizer and pesticide use, represent the most important challenges society faces in the coming years. To this end, agricultural practice is moving towards a more sustainable and environmentally friendly approach. Modern strategies increasing agricultural productivity emphasize the importance of microbial inoculants in realizing their objective. Application of the microbial products is current closely reviewed and product lists are published [1,2,4,5,6].

Because of the complexity of microorganism associations with their hosts, further research is needed to comprehend the fundamental mechanisms underlying plant growth promotion by plant growth-promoting bacteria (PGPB). Plants have developed a variety of mechanisms to cope with the negative impact of biotic and abiotic factors. The ability to communicate and establish a stable relationship between plants and microorganisms clearly increases the capacity of plants to cope with stress, and also facilitates their growth and development [5]. Interactions between host plants and microorganisms can occur through endophytic, epiphytic or rhizosphere processes. Plant growth-promoting endophytes (PGPE) are beneficial agents, because they create a favorable environment for plant growth and development. The term “endophyte” is derived from “endon” meaning within, and “phyton” meaning plant. Recently, the definition of the term “endophyte” has been modified several times. According to Hardoim et al. [7], endophyte should refer to plant interior habitats, regardless of the function and result of the association with the plant. On the other hand, Le Cocq et al. [8] and van Overbeck and Saikkonen [9] proposed that endophytes should be defined as “microbes which colonize internal plant tissues for at least parts of their life cycle without causing disease symptoms under any known circumstances”, in other words, without causing any visible detriment to the host plant.

Based on the metabolic and physiological properties of bacteria, their diversity can be determined as well as PGPB that have a positive impact on the plant growth can be selected. Polyphasic taxonomy based on different types of data from phenotypic, genomic and phylogenetic analysis is currently most commonly used to identify microorganisms [10]. Phenotypic analysis allows preliminary classification of bacteria into the genus and even to the species level. Currently, more attention is focused on application of a semi-automated phenotypic/physiological BIOLOG^TM^ system, which allows a standardized and easily reproducible identification. This system is connected with a computer database and includes a wide range of carbon substrates used by microorganisms. The tetrazolium dye, reduced to a purple formazan, functions independently of the specific structure of the electron transport chain and is able to detect the ability to metabolize any substrate [11]. The test has been used to identify, among others, bacterial pathogens from clinical samples [11,12] and bacteria from environmental samples [13].

Many studies have shown that endophytes regulate plant growth and development. Endophytic bacteria are examined for direct and indirect plant growth-promoting (PGP) mechanisms. Direct growth mechanisms include: solubilization of mineral nutrients like phosphorus, zinc and potassium, biological N_2_ fixation, production of different types of phytohormones, such as auxins, cytokinins, and gibberellins, sequestration of iron by siderophores, and production of aminocyclopropane‑1‑carboxylic acid (ACC) deaminase and volatile growth stimulants, i.e., acetoin and 2,3-butanediol. Indirect plant growth-enhancing mechanisms include the production of: antibiotics, siderophores, hydrogen cyanide (HCN), ammonia and lytic enzymes, as well as induced systemic resistance (ISR) [1,14,15]. Many endophytic bacteria belong to the genera *Pantoea*, *Pseudomonas*, *Serratia*, *Klebsiella* and *Herbaspirillum*, which have been isolated from tissues of various plants, such as maize, soy, rape, wheat, onion, cauliflower and cucumber, providing the ability to dissolve non-organic phosphates, produce proteases and lipases, as well as cellulase and pyoverdines (siderophore) [16]. In addition, endophytic isolates belonging to the genera *Enterobacter*, *Rahnella*, *Rhodanobacter*, *Pseudomonas*, *Stenotrophomonas*, *Xanthomonas* and *Phyllobacterium* were shown to produce the phytohormone IAA (auxin), and had the ability to grow in nitrogen-free medium [17,18].

Bacterial endophytes represent a large reservoir of undiscovered genetic and functional diversity. Microbial endophytes differ among plant species, and their diversity is influenced by biotic and abiotic factors, such as the plant host species, the growth stage of the host, tissue type, soil type, latitude, climate, and agro-technical management [19,20,21,22]. Currently, the microbial endophytes associated with crops have been gaining attention, because sustainable agriculture and healthy food production are urgent needs in the 21st century. An understanding of the mechanisms that promote plant growth, as well as the interactions of the microbes with the crops, can be helpful in selecting those species and conditions that will have a maximum biostimulatory effect.

Even so, there is a lack of comparative studies on the promotion of growth and functional characteristics of endophytic bacteria isolated from crop plants and weeds at the same site. Such tests may contribute to the inoculation of effective bacteria on a non-host plant. In the current study, the concepts of Le Cocq et al. [8] and van Overbeck and Saikkonen [9] were adopted, that endophytes are non-pathogenic organisms that live inside plant tissues. The main goal of this current study was to screen and characterize PGPB strains in vitro, on the basis of their association with plant growth promotion, i.e., the ability to produce IAA and siderophores, to solubilize phosphorus, and to sequester iron. This study also focused on the phenotypic characterization of endophytic bacteria obtained from the roots and stems of crops and wild plants, representing both different species and phyla, identified by sequencing 16S rRNA gene fragments in a previous report.

## 2. Results

### 2.1. Phenotypic Profiling and Identification of Endophytic Bacteria Using Biolog^TM^ GEN III MicroPlates

GEN III software allowed for the classification of all tested isolates to the species level, based on phenotypic tests and chemical sensitivity. Results of identification on GEN III plates were compared to those based on the sequencing of the 16S rRNA gene. This comparison indicated a degree of uncertainty regarding the identification of endophytic bacteria isolates. This could be observed in case of the metabolically less-active isolates, for example, ER1, VR2 and VS3, based on average well color density (AWCD). Moreover, the two standard strains were not represented in the microbial identification database for the Biolog System. However, 18 isolates (78%) were correctly identified at the genus level and 11 at the species level (Table 1). The application of the GEN III MicroPlate in double reiteration allowed for the comparison of the functional diversity of all the endophytic bacterial strains.

**Figure 1:**
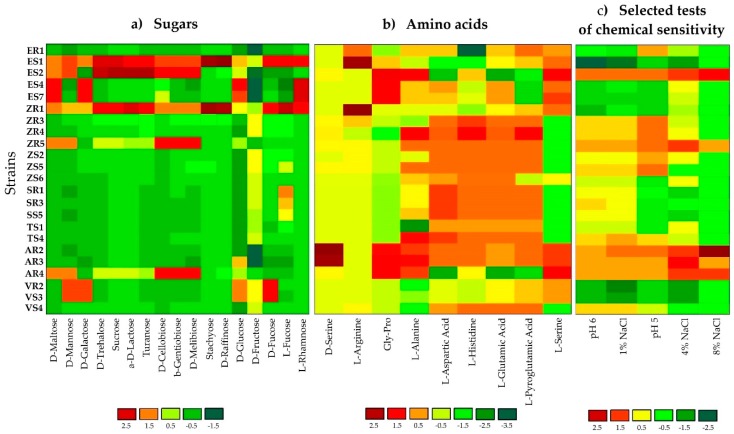
Heatmap of 23 strains showing physiological profiles for (**a**) sugars, (**b**) amino acids and (**c**) selected tests for chemical sensitivity after 120 h of incubation. The relative utilization of selected substrates is depicted by color intensity, based on the legend next to the figure. The highest consumption can be identified by a red color and the lowest consumption by a green color.

GEN III MicroPlates, AWCD and diversity index analysis, allowed for identification and comparison of the metabolic profiles of endophytic bacteria selected from various plant hosts (Table 1 and Figure 1). The utilization profiles of substrates and chemical sensitivity assays for tested isolates revealed a broad variability (Figure 1). Strains ES2 (*Stenotrophomonas maltophilia*), ZR1 (*Novosphingobium resinovorum*), ZR3 (*Delftia acidovorans*) and ZR4 (*D. acidovorans*) showed the highest metabolic activities on the tested substrates. Whereas, ES4 (*Brevundimonas* sp.), ES7 (*Brevundimonas* sp.), and ER1 (*Comamonas koreensis*) were characterized by the lowest utilization of all carbon sources. Analyzing strains at the genus level, bacteria classified as *Stenotrophomonas* sp. had the strongest metabolic activity (AWCD_590 nm_ = 133.28). Notably, maize tissues contained the most active catabolic strains (AWCD_590 nm_ = 126.21). The diversity of metabolic activity of endophytic strains was calculated by functional diversity indices. The Shannon diversity (H′) and Shannon evenness (E) indices of tested bacteria are shown in Table 1. The diversity of metabolic activity based on the Shannon diversity and Shannon evenness indices was 3.75–4.34 and 0.90–0.98 respectively, indicating a high metabolic diversity for the endophytic bacteria in the current study (Table 1). The highest values for the Shannon diversity and evenness indices occurred in strain ZR1, followed by ES1, indicating that these bacteria exhibited a high metabolic potential and high metabolic biodiversity of carbon source utilization. In the analysis of phenotypic/physiological profiles, values for indices of functional diversity (AWCD, H′ and E) were different in almost all endophytic bacteria, even within the same genus, e.g., *Delftia* strains ZR3 and SR3, as well as *Stenotrophomonas* strains ZR5 and ES. The utilization profiles of selected substrates and chemical sensitivity assays for the tested isolates also revealed a broad variability. A comparison of the metabolic profile of tested isolates based on the utilization of sugars (Figure 1a), amino acids (Figure 1b) and selected tests of chemical sensitivity (Figure 1c) were documented as a heatmap. This analysis illustrated differences on consumption of the same substrates. The level of substrate utilization of all the strains was highest on D-fructose and D-Glucose for sugars, followed by L-histidine and L-Aspartic Acid for amino acids. By contrast, D-raffinose and stachyose were the lowest utilized sugars, and D-serine was the lowest metabolized amino acid.

Sensitivity to salinity (1%, 4% and 8%) and pH (5 and 6) by endophytic isolates was also determined on Biolog GEN III MicroPlates (Figure 1). Most endophytic isolates were resistant to low salinity (1% and 4%); however, only AR2, AR3, AR4, ES2, SR3 and ZR5 strains were able to grow at 8% NaCl. In general, the number of isolates showing resistance to salinity decreased with increasing NaCl concentration. In addition, all of the endophytic isolates were able to grow at pH 6; whereas, only 74% of strains were able to grow at pH 5.

### 2.2. In Vitro Screening of Bacterial Isolates for Potential Plant Growth Promoting (PGP) Activities

#### 2.2.1. Colorimetric Analysis of Indole-3-Acetic Acid (IAA)-like Compounds Production

Synthesis of IAA-like compounds were examined using Salkowski’s reagent. The change in color was clearly visible in the first minutes of the experiment at the highest concentration of IAA, increasing in intensity for a period of 30 min. The selected bacterial isolates were characterized by their abilities to synthesize IAA, both in the presence and absence of tryptophan (L-Trp; Table 2). Almost all isolates were able to produce IAA, with concentrations dependent on the strain, genus of bacteria, host plant and presence of the amino acid precursor. For 22 isolates, aside from strain AR3, Luria- Bertani (LB) medium with L-Trp was more suitable for IAA synthesis compared to LB without L-Trp. The addition of L-Trp into bacterial culture medium increased the efficiency of IAA biosynthesis from 0.74 (VS3) to 18.25 (TS4) times. All selected isolates showed IAA-producing ability in the range of 0.13 (AR3) to 22.51 µg IAA/mL (TS4), in liquid culture supplemented with L-Trp. In LB medium without L-Trp, AR4 and ZR1 produced the highest and lowest amounts of IAA, respectively. Only AR3 showed no ability to synthesize IAA in medium without L-Trp. Most strains of endophytic bacteria classified to the genus *Delftia* (ZS2, SR1 and TS4) showed elevated synthesis of IAA (above 10 μg IAA/mL), with the highest levels being recorded by *S. maltophilia* (ES2 and AR4).

#### 2.2.2. Qualitative and Quantitative Production of Siderophores

**Figure 2:**
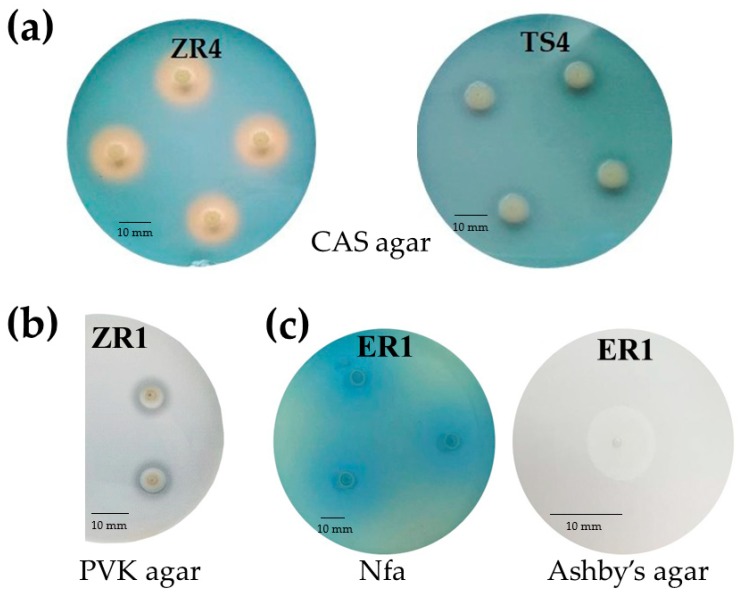
(**a**) The production of siderophores based on the formation of an orange zone around colonies on CAS agar after 72 h of incubation. Strain ZR4 presented positive production of siderophores, while strain TS4 show no production of siderophores. (**b**) Phosphate solubilizing by the ZR1 strain on Pikovskaya agar after 120 h of incubation. (**c**) Oligonitrotrophic bacteria ER1 on Nitrogen-free (Nfa) agar medium and Ashby’s mannitol agar (in zoom) after 72 h of incubation.

Siderophores production by endophytic bacteria was observed by formation of an orange-colored zone around bacterial colonies on chrome azurol S (CAS) agar plates. Production of siderophores was estimated by the size of this zone. The tested isolates were classified to two groups, those that grew, but did not form a halo zone around the colonies, and those that showed growth as well as a small to large orange zone (Figure 2a). ZS2 (*D. acidovorans*), ZS5 (*D. acidovorans*), VS4 (*D. acidovorans*) and ER1 (*C. koreensis*) showed the largest siderophores production on CAS agar plates (Table 2).

All 23 endophytic bacteria isolates were examined quantitatively for the production of siderophores; however, only 14 bacterial isolates (61%) were positive for siderophores, showing different intensities of the orange zone. The amount of siderophores produced by all the 23 strains was compared with the traditional method on agar plate (Table 2). The amount of siderophores produced by the different endophytes varied between the different isolates. About 74% of isolates were able to produce siderophores in liquid medium, ranging from 3.45 (AR2) to 81.62 (ZS2) percent siderophores units (psu). The two strains classified as *D. acidovorans* (ZS2 and ZS5) produced the highest levels of siderophores. The next two highest producers were *C. koreensis* and a further isolate of *D. acidovorans* (ER1 and VS4). By contrast, a negligible amount of siderophores (<3.50 psu) was produced by endophytic strain AR2 (*Coliomonas pratenisis*). The ability of bacterial strains to synthesize catechol siderophores (CTS) was determined by the method of Arnow [23], while hydroxamate siderophores (HTS) were detected by the assay of Csáky [24] (Table 2). The highest production of HTS was observed for a strain of *Achromobacter* (AR3; 2.11), followed by *D. acidovorans* ZR4 (2.04); whereas, the lowest level was recorded for ZS6, a strain of *Delftia*. Different amounts of CTS were identified in 12 strains of endophytic bacteria. The highest level of CTS was found in strain ES7, a *Brevundimonas* sp. isolated from stem tissue of horsetail (1.93). By contrast, the lowest level of CTS production was observed in *Rhizobium* isolate ES1 (0.01). Nine tested isolates demonstrated the ability to produce both HTS and CTS at different concentrations.

#### 2.2.3. Phosphate Solubilization

The phosphate solubilization test was performed by observing halo formation around bacterial colonies after incubation for seven days at 28 °C (Figure 2b; Table 2). Only 13% of the isolates were able to solubilize tricalcium phosphate in vitro in solid medium. Two distinct groups were formed according to the classification of Berraquero et al. [25]. One isolate was classified as a high solubilizer, two were classified as medium solubilizers, while no isolate was classified as a low solubilizer. Isolate ZS6 (*Delftia* sp.) showed the largest solubilization index (4.1; Table 2). Twenty endophytic isolates grew on solid medium containing insoluble phosphate; however, they did not form a halo zone, and were therefore unable to solubilize phosphate in this form.

#### 2.2.4. Oligonitrotrophic and Nitrogen-Fixation Screening

Oligonitrotrophic bacteria were preliminary screened on nitrogen-free (Nfa-Nitrogen-free agar) medium, containing bromothymol blue (BTB) as an indicator, and on Ashby’s mannitol agar. All selected endophytic bacteria showed the ability to grow on these two N-free solid media. On Nfa agar medium, the studied strains formed a characteristic blue zone around the colonies (Figure 2c). The nitrogen fixing capacity of selected endophytic strains was assessed based on the presence of the *nifH* gene. The results revealed that only one (AR2-*Collimonas pratensis*) of the 23 strains showed no *nifH* gene amplification with the size of the fragment about 390 bp. The presence of the evolutionarily conserved *nifH* gene showed that the 22 isolates have the ability of nitrogen fixation (Figure S1).

The efficiency of N_2_ binding by bacterial strains was determined based on the increase in total N content in bacterial cultures carried out on liquid nitrogen-free medium after 24 h, 48 h and 72 h of incubation at 28 °C (Table 3). Most strains effectively fixed N_2_. Strains ZR4 and AR2 after 24 h growth did not show the ability to fix atmospheric nitrogen. The effectiveness of the remaining strains after 24 h was from 13.00 (SR3 strain) to 34.85 mg of N/g of carbon (TS4). In 48 h incubation, the ZR4 strain showed the ability to increase nitrogen during cultivation, while the AR2 strain remained inactive. Overall, the amount of bound nitrogen increased as the incubation time increased. After 72 h of incubation, the most effective atmospheric nitrogen assimilators were: TS4 (*Delftia acidovorans*) and AR3 (*Achromobacter xylosoxidans*), and the less effective strain: ZR4 (*Delftia acidovorans*). The AR2 (*Collimonas pratensis*) strain did not show atmospheric nitrogen binding capacity. After 72 h of incubation into bacterial culture medium, the efficiency of nitrogen fix increased from 0.87 (for AR4) to 1.67 (for ES1) times. The positive (mostly >0.9) correlation between incubation time and efficiency was observed. The only exception was for the AR4 strain, where no such correlation was observed. High efficiency results from high nitrogen levels—the highest in culture of the TS4 strain, were maintained from the first day of culture incubation.

### 2.3. Statistical Analysis

**Figure 3:**
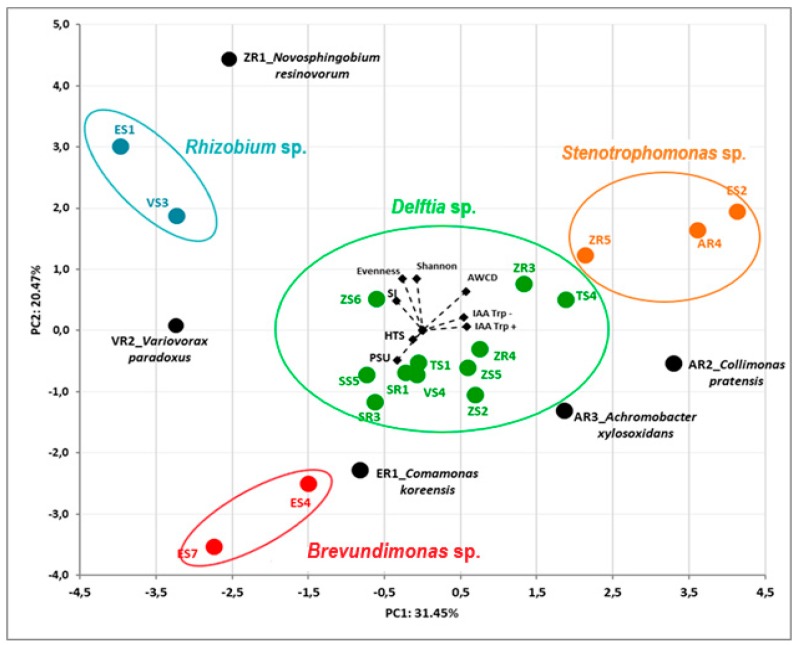
Biplot diagram of Principal Component Analysis (PCA), describing plant growth-promoting activities (IAA, HTS, PSU, SI) and biodiversity (Biolog GEN III) indices (AWCD, Shannon, Evenness) of 23 bacterial endophytes isolated from plants. Strains classified based on the nucleotide sequence of their 16S rRNA genes to the same genus are indicated in different colors.

The dependence between all the endophytic bacteria and their properties was assessed by principal component analysis (PCA). The first two principal components (PCs) accounted for 31.45% and 20.47% of the total variance of the tested bacterial isolates. The PCA allowed us to separate the bacterial endophytes based on data from sequencing their 16S rRNA genes (Figure 3). The AWCD value was positively related for strains TS4, ZR3, ZR5 AR4 and AR2, whereas it was negatively related for strains ER1, ES4 and ES7. PCA of bacterial endophytes showed a strong separation based on their genus, resolving four main groups of strains and four individual isolates (Figure 3).

**Figure 4:**
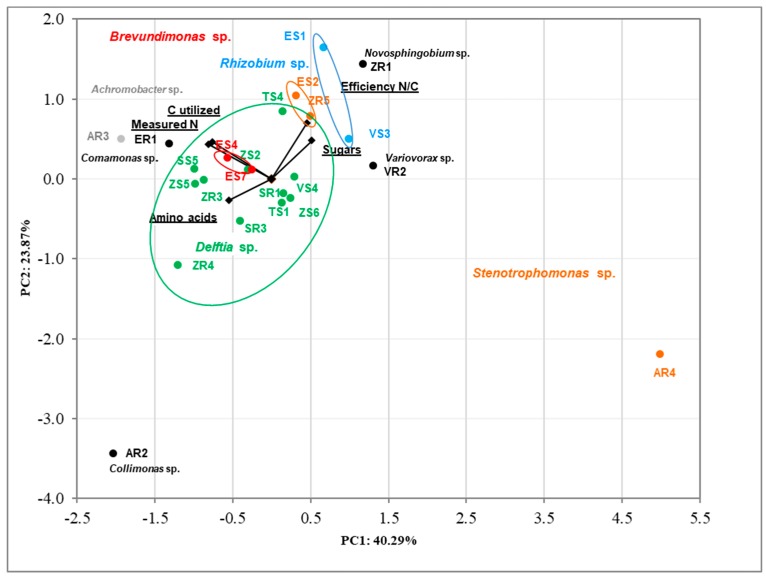
Biplot diagram of Principal Component Analysis (PCA), describing amino acids and sugar utilization, efficiency of nitrogen fixation and nitrogen produced, carbon utilized for 23 bacterial endophytes isolated from plants. Strains classified based on the nucleotide sequence of their 16S rRNA genes to the same genus are indicated in different colors.

The correlation between tested bacteria using analysis of produced nitrogen, carbon utilized, efficiency N/C, and sugars and amino acids utilization was determined via Principal Component Analysis (PCA) (Figure 4). The results were in accordance with previous PCA. The clear cluster based on genus bacteria was noted. The first two dimensions of PCA explained 64.16% of the total variation, with principal component 1 (PC1) accounting for 40.29% and principal component 2 (PC2) accounting for 23.87% of the variance. The AR2 and AR4 strains are clearly different from the others.

On the basis of the PCA, in both those based on selected PGP activities and biodiversity indices (Figure 3), as well as in those based on amino acids and sugars utilization and efficiency of nitrogen fixation (Figure 4), the same trend was observed. In the current study, the analyzed complex of properties responsible for promoting plant growth by the endophyte’s was determined by their taxonomic affiliation.

The data received by PCA were confirmed by correlation analysis (Table 4), in which all results obtained for the tested bacteria isolates were included. Spearman’s rank correlation coefficient with the Bonferroni correction was used for testing correlations between PGP activities and functional diversity of endophytes. The Spearman correlation matrix demonstrated that values of AWCD had a significantly positive correlation with the Shannon diversity index, pH 6 and pH 5, as well as 1% salinity. A strong positive correlation was observed between IAA-like compounds Trp- and Trp+, as well as between psu and CTS values.

## 3. Discussion

The endosphere, which is inhabited by numerous bacteria that potentially promote plant growth, represents a valuable resource for sustainable and ecological agriculture. Biostimulators and bacterial inoculants are environmentally friendly, being an alternative to the large-scale use of commercial, synthetic chemicals in agriculture, such as fertilizers and pesticides [26]. There are several common steps to creating an effective biofertilizer, including: the selection of suitable plants for the isolation of microbiological components of the biopreparation, proper isolation of the endophytic microorganisms, culturing of these isolates under laboratory conditions, determination of the systematic affiliations of the endophytes, and in vitro testing of strains for plant growth stimulation [27]. Before commercializing a product, the most efficient bacterial strains need to be analyzed on plants in phytotrons, greenhouses and field experiments, as well as in natural soil and climate conditions.

### 3.1. Phenotypic Profiling and Identification of Endophytic Bacteria Using Biolog^TM^ GEN III MicroPlates

The tested strains in the current study were isolated from internal tissues of two separate parts of six species of vascular plants, stems and roots. The isolated strains represented nine genera *Delftia* (11), *Stenotrophomonas* (3), *Rhizobium* (2), *Brevundimonas* (2), *Variovorax* (1), *Achromobacter* (1), *Novosphingobium* (1), *Comamonas* (1) and *Collimonas* (1) belonging to one phylum Proteobacteria. The bacterial communities on and inside various plant organs are defined by relatively few bacterial phyla, including, Actinobacteria, Bacteroidetes, Firmicutes, and especially Proteobacteria. Many studies have indicated that bacteria belonging to the phylum Proteobacteria dominate both the rhizosphere and plant tissues, with members being consistently enriched in plant roots compared to the surrounding soil biome [28,29,30]. Partida-Martinez and Heil [31] distinguished four classes of endophytes, and among them, three classes of fungi and only one class of bacteria—diazotrophs, belonging to the order *Rhizobiales*. All isolated bacterial endophytic strains tested by us belong to phylum Proteobacteria, like bacteria of the genus *Rhizobium* (or from order *Rhizobiales*). Our research confirmed that these tested endophytic strains can be included in the fourth class of endophytic diazotrophs. Huang found that for *Allium tuberosum,* they accounted for almost 40% of the bacterial community in the rhizosphere, 14.75% in the leaves and 21.04% in roots, with Cyanobacteria dominating in the endosphere (83.42% in leaves and 75.44% in roots) [32].

The first very important step in the development of a microbial-based biopreparation is the identification and characterization of the bacteria. Currently, most microorganisms are identified using the 16S rRNA sequencing technique. In a previous study, this technique was used to differentiate endophytic bacteria from crops, as well as wild plants [19]. The identification carried out on phenotypic profiles and metabolic activities in this current study, using the Biolog GENIII MicroPlate-based technique, gave different results for five root isolates compared with the previous identification. Misidentification at the genus level was confined to less metabolically active isolates. Misidentification may occur because microorganisms present with uncommon phenotypes changed under environmental conditions [33]. Identification of bacterial isolates with the Biolog system showed high accuracy, with a total score of 78%. For more active biochemical bacteria, the system demonstrated a good capacity for establishing identification, e.g., at a similarity level of 0.76 for strain ZS2 (based on the Biolog database). The Biolog GEN III MicroPlate™ test might provide valuable confirmation at the species level, based on the analysis of phenotypic profiles; however, it might be inappropriate as a single identification method. Identification based on 16S rRNA gene sequencing provides improved resolution and is not influenced by phenotypic variation or changing environmental conditions. Similar results were obtained by Morgan et al., in which 16S rDNA gene sequencing was shown to have the highest percent accuracy, with 90.6% correct identifications, while the Biolog system identified 68.3% of the isolates correctly [34]. Even so, in addition to isolate identification, the Biolog system allows for the determination of substrate utilization profiles and chemical sensitivity assays. It could be assumed that strains extensively metabolizing a wide range of substrates and showing more adaptation to high salinity concentrations and acidic pH could be more adapted to changing environmental conditions. Notably, strains isolated from the tissues of wild plants (AR4, ES2, AR2, AR3) were more adapted to high concentrations of salinity (4% and 8%), as well as acidic pH. In the presence of a stress factor such as salinity, plant resistance to this abiotic factor may be additionally induced, i.e., the overall resistance of the plant may increase. The cross-tolerance between stresses exists. The important role in salinity tolerance is played by ROS (reactive oxygen species) substances generated in first step of resistance pathways. Mechanisms of salinity tolerance in plants was extensively reviewed by Tuteja [35]. Mechanisms of salinity tolerance involve sequestration of Na and Cl in vacuoles of the cells, blocking of Na entry into the cell, Na exclusion from the transpiration stream, SOS (salt overly sensitive) pathways, transcription factors, mitogen-activated protein kinases, production of glycine betaine, prolin, and ABA (abscisic acid) hormone. Salinity stress has a significant effect on agriculture because it negatively impacts the growth and development of plants. Moreover, there is also an alternative way to combat high soil salinity. The highly salt-tolerant bacteria, along with plant growth-promoting properties, would be beneficial for use in the mitigation of salt stress to make cultivation possible in saline agriculture lands [36].

### 3.2. In Vitro Screening of Bacterial Isolates for Potential PGP Activity

In the current study, a dominance of *Delftia* spp. was observed. The pronounced domination of diazotrophic Proteobacteria (72–96%), including strains from the genus *Delftia* (10–38%), has also been confirmed in a widespread epiphytic orchid, *Dendrobium catenatum,* in a different climatic and geographical zone in China [37]. The ability of *Delftia* strains to fix atmospheric nitrogen, and produce phytohormones, siderophores, antifungal compounds (heterocyclic N) and 1-aminocyclopropane-1- carboxylate (ACC) deaminase, solubilize phosphate and perform sulfur oxidation, was reported by Banach et al. and Brana et al. [38,39]. *Delftia* strains have been reported as plant growth promotors of many plants, including rice, tomato and some legumes [40,41,42,43,44]. Morel et al. [40,41] reported that strain *Delftia* sp. JD2 has the ability to detoxicate Cr-contaminated soils. Moreover, this strain showed plant growth-promoting activity in gnotobiotic conditions. The next study reported by the same scientific group [42] confirmed that other *Delftia* strains 3C and 6C, are resistant to heavy metals Cr(VI) and Pb(II) and promote the growth of clover (*Trifolium repens*) in greenhouse conditions. The results of the above reports suggested that *Delftia* sp. could be good candidates for the bioremediation of contaminated environments and the ecological biofertilizers. The study of Han and co-workers [43] on the efficacy of *Delftia tsuruhatensis* strain HR4, isolated from the rhizoplane of rice (*Oryza sativa* L., cv. Yueguang) to inhibit main rice pathogens, confirmed their antagonistic effects. Moreover, strain HR4 also showed a high nitrogen-fixing activity. The potential of bacteria genus *Delftia* to inhibit the growth of tomato (*Lycopersicon esculentum*) pathogens *Fusarium*, *Sclerotium*, *Pythium* and *Rhizoctonia* was also noted [44].

*S. maltophilia* strains synthesized the highest concentrations of IAA-like compounds, both in the presence (about 20 μg IAA/mL) and absence (about 4 μg IAA/mL) of L-Trp. According to Ambawade et al. [45], the endophytic *S. maltophilia* BE25 strain isolated from the roots of Banana (*Musa* spp.) produced 39 μg IAA/mL, while Brigido et al. [46] showed that strains of the genus *Stenotrophomonas* isolated from chickpea roots produced only low levels of IAA. Shi et al. [47] observed an increase in the growth of sugar beet by inoculation *Bacillus pumilus* synthesizing IAA at the level of about 10 µg/mL (incubation time 24 h) and *Acinetobacter johnsonii* producing 40 µg/mL of IAA (144 h). Our study provided different results because the strains selected for plant inoculation produced small amounts of IAA-like compounds (0.56 µg/mL for strain VS3) to concentrations at a medium level (22.51 µg/mL for strain TS4).

Despite the high potential to promote plant growth, it should be added that *Delftia acidovorans* and *Stenotrophomonas maltophilia* strains can cause human diseases. However, these infections most commonly occur in hospitalized or immunocompromised patients. It needs to be highlighted that opportunistic pathogens of environmental origin could be damaging for agriculture and thus for human health. Therefore, there is a need to establish effective protocols to distinguish harmless from harmful strains [48,49]. Before establishing a future field experiment to assess the effectiveness of promoting plant growth and development by bacteria belonging to *Stentrophomonas maltophila* and *Delftia acidovorans*, additional analyses were performed. Research will be conducted to confirm that the selected strains are harmless for biotechnological applications and without human health risks. First of all, bacterial growth at 37 °C (the human body temperature) will be checked.

Generally, microorganisms isolated from the rhizosphere, rhizoplane and tissues of various crops are more active in producing auxins than those from root-free soil [50,51,52]. L-tryptophan is considered a physiological precursor of auxins in higher plants and microbial biosynthesis. Plant roots contain L–Trp, which can be consumed by microorganisms as a precursor for IAA production [53]. The endogenous IAA level occurs highest in the apical young zone of stems and in the apical and basal part of roots. Moreover, studies on the meristem of *Arabidopsis* suggest that such endogenous gradient of IAA plays an integral role in morphogenesis [54]. The results of experiments carried out in the early 90 s on eyelash (*Lemna gibba*), suggested the possibility of functioning other IAA biosynthesis pathways, independent of tryptophan [55]. These suggestions were confirmed in the study [56,57]. Michalczuk et al. [58], analyzing changes in IAA concentration in carrot cell cultures, showed that even in the same plant, IAA can be synthesized in a tryptophan-dependent or independent pathway. It should be emphasized that there is no direct relationship between the level of endogenous IAA and exogenous IAA supplied to the plant as a synthetic compound or synthesized by endophytic microorganisms. Olantuji et al. [59] reviewed the plant’s regulation of the endogenous auxin concentrations by biosynthesis, catabolism and conjugation. In a response to environmental cues, root growth is adapted through the modulation of endogenous auxin levels, and the establishment of auxin gradients in the root requires the interplay of local auxin biosynthesis, transport, perception and signaling. Ribnicky et al. [60] explained the complicated relationship between exogenous and endogenous IAA levels. Exogenous IAA can be rapidly metabolized to form inactive conjugates or possibly because it mediated a decrease in endogenous IAA concentrations by an apparent feedback mechanism. The presence of exogenous auxins did not affect tryptophan labeling of either the endogenous tryptophan or IAA pools. This suggested that exogenous auxins did not alter the IAA biosynthetic pathway. Hermosa et al. [61] presented, in a comprehensive review, a scheme of IAA influence on the level of plant hormones/signaling substances, which clearly showed that the concentration of IAA and auxin signaling in a plant is positively correlated with the level of ethylene and ABA and the latter reduces

*Stenotrophomonas* and *Delftia* strains not only have the ability to synthesize phytohormones, but also produce other metabolites that stimulate plant growth, such as siderophores, as well as fixing atmospheric nitrogen and solubilizing phosphate [45,62]. Poor soil fertility is one of the major constraints for crop production. Nitrogen (N) is the key plant nutrient required for plant growth but at the same time, is the most limiting nutrient for increasing crop productivity. Therefore, there is a need to identify diazotrophic inoculants as an alternative or supplement to N-fertilizers for sustainable agriculture [63,64]. Isolation and screening for potential diazotrophic bacteria are crucial steps in research on biofertilizer. For example, an in vitro screening procedure (growth on Nfb agar medium and Ashby’s mannitol agar, Kjeldahl method) and its combinations provides rapid and repeatable results [64]. In addition, genetic analysis of the *nifH* gene—one of the most important genes in the biological nitrogen fixation system, can be an important confirmation of the ability of the tested strains to bind to nitrogen [65]. In our research, conventional methods proved valuable in the initial stages of the experiment and confirmed that all tested strains could be potential nitrogen fertilizers. Additional genetic analyses based on the amplification of the *nifH* gene were needed, which confirmed the diazotrophy of the 22 from 23 isolates tested.

Many recent reports indicate that endophytic microorganisms can simultaneously regulate hormone management, plant defense and the supply of iron and other elements, as these processes are very closely related [66]. Because iron (Fe) availability to plants is low [67], it is very important for plants using Strategy I root response to Fe deficiency (dicots and non-grass monocots), as well as those using Strategy II (grasses), support the supply of Fe by PGPB [66,68]. Mechanisms alleviating Fe deficiency and those involved in ISR (Induced Systemic Resistance) against pathogens and insects might be closely interconnected [69,70]. Endophytic bacteria are now being developed for their role in increasing plant growth, through their ability to produce siderophores that bind available forms of iron (Fe^3+^), making it unavailable to phytopathogens, and hence protecting plant health [71]. In the current study, strains of the genus *Achromobacter* were the strongest producers of HTS, *Brevundimonas* CTS, while *Comamonas* strains synthesized the strongest Fe-chelators (CAS test). Arora and Verma [72] noted that the endophytic strain RB1 *Pseudomonas aeruginosa* synthesizes siderophores at a level of 44.44 psu. Moreover, Gosh and co-authors [73] reported that *Bacillus subtilis* exhibited 65 percent of siderophore production unit. Strains isolated in our study are much more active, for example, the strains of bacteria from the genus *Delftia* ZR4, ZS2, ZS5, SR1 and VS4 synthesize siderophores at a level above 60 psu.

### 3.3. Principal Component Analysis (PCA) of Isolates’ Features

Analysis of various properties considered to be favorable for the promotion of plant growth for 23 endophytic bacterial strains subjected to PCA clearly indicate that these features were dependent on the genus of the bacterium, as identified by 16S rRNA gene sequencing, and not on the plant species or part of the plant from which the tested bacteria were isolated. In recent years, Jacoby et al. [74] reported an important issue, that plants shape microbiome structures, most probably by root exudates. Consequently, it is believed that this plant decides not only which of the microorganisms to colonize it, but also what characteristics will be available to these microorganisms. The results obtained by us, subjected to statistical PCA analysis, clearly indicated that the complex features responsible for promoting plant growth at the endophyte’s disposal, is not determined by the plant, but it is a set of properties characteristic for the genus of endophytic bacterium. This is confirmed by numerous experimental works, the results of which were presented in the review by Gouda et al. [75]. This comparison showed that strains belonging to one genus, e.g., *Azospirillum, Azotobacter, Bacillus, Paenibacillus, Pseudomonas*, possessing features characteristic for appropriate genus, were isolated from various, often very distant, plant species, and were also effective in promoting the growth of these different plant species. Santoyo et al [76] discussed extensively bacterial PGP endophytes, and indicated that plants representing different plant species may be the host for the same strain. Bai et al. [77], who studied the microbiome of the rhizosphere, roots and leaves of *Arabidopsis thaliana*, came to very similar conclusions to ours, regarding the dependence of features and properties on taxonomic affiliation. After statistical treatment of results using Principal Coordinates Analysis (PCoA) of functional distances, it revealed a clear clustering of genomes on the basis of their taxonomy, but only a limited separation of genomes on the basis of their ecological compartment. Thus, they found that both phylogenetic and functional diversification of the genomes is strongly driven by their taxonomic affiliation, and weakly by the ecological niche.

The current study provided hopeful results that will contribute to the selection process of the most promising for plant growth endophytic bacteria. The obtained results indicate that isolates from different plant species can be used in fertilization, stimulation or protection of other species, because different plants are inhabited by endophytes from the same genera and species. In addition, it provided a basis for further insightful research on bacterial phytohormones and the enzymes regulating plant phytohormone pathways, such as ACC deaminase, and on crosstalk between phytohormones and induced resistance in plants.

## 4. Materials and Methods

### 4.1. Sample Collection

Twenty-three strains of endophytic bacteria were isolated from four different crops and two wild plants. Bacteria were selected from the roots and stems of healthy and mature plants: *Zea mays L. (maize), Vicia faba L. (broad bean), Secale cereale L. (rye), Triticum aestivum L. (wheat), Arctium lappa L. (burdock) and Equisetum arvense L. (horsetail).* A description of the bacteria isolation process, and the results of their identification based on 16S rRNA gene sequencing, were presented in a paper by Woźniak et al. [15]. All isolated strains were stored at 4 °C on tryptic soy agar (TSA) (Difco Laboratories, Inc., Franklin Lakes, NJ, USA), and as lyophilized cultures in glass ampoules.

### 4.2. Phenotypic Profile and Identification of Endophytic Bacteria Using Biolog^TM^ GEN III MicroPlates

The pure biological cultures were identified and characterized by the Biolog GEN III system (Biolog Inc. Hayward, CA, USA), following the manufacturer’s instructions. This method allowed the establishment of a metabolic profile for specific microorganisms, i.e., a “phenotypic fingerprint”. The GEN III MicroPlates™ provided micro-testing of bacteria, assessing the ability to metabolize 71 carbon sources and containing 23 chemical sensitivity assays. The GEN III plates contained tetrazolium redox dye, which was used to calorimetrically indicate positive reactions. The inoculation procedure was based on the original GEN III microplate method (Biolog^TM^) according to the manufacturer’s protocol. Bacterial colonies were transferred to inoculating fluid A (IFA) with a sterile cotton swab to generate bacterial cell suspensions, the transmittance of which were adjusted between 90% and 98% using a turbidimeter (Biolog^TM^). Then, 100 µL of the cell suspension was dispensed into each well. The absorbance of each well of the inoculated microplates was read at 590 nm on a Biolog MicroStation™, at 24 h intervals over six days. The analysis was carried out as two biological replicates for each strain. The most consistent readings came from six-day-old Biolog plates, and these data were used in the analyses. Results were captured and analyzed based on an extensive species library in the Biolog GEN III database [78].

### 4.3. In Vitro Screening of Bacterial Isolates for their Potential PGP Activities

#### 4.3.1. Colorimetric Analysis of IAA-like Compounds Production

IAA-like compounds production by selected strains, both in the presence and absence of 0.1% L-Trp, was determined as described by Glickmann and Dessaux, using Salkowski’s modified reagent R1 (12 g FeCl_3_ in 1000 mL 7.9 M H_2_SO_4_) [79,80,81,82]. All strains were incubated in triplicate in Luria Broth (LB) at 28 °C in for 48 h on a rotary shaker. Then, culture supernatants were collected after centrifugation at 10,000 g for 10 min. Non-inoculated control medium was kept for comparison. One milliliter of culture supernatant was mixed with 1 mL of Salkowski’s reagent R1 and allowed to react in darkness at room temperature for 30 min. The development of a pink color indicates the presence of IAA. The quantity of IAA produced was measured using a VarianCary 1E UV-Visible Spectrophotometer against a standard curve of IAA (Sigma-Aldrich, St. Louis, MI, USA).

#### 4.3.2. Qualitative and Quantitative Production of Siderophores

Qualitative production of siderophores was assessed by the ‘universal’ method described by Schwyn and Neilands [83]. Strains were spotted on agar plates containing the dye CAS. The formation of an orange zone around a bacterial colony on the blue agar was considered positive for siderophores excretion [84]. Quantitative estimation of siderophores production was performed by the microplate method described by Arora and Verma [72], using a microplate reader (VarianCary 1E UV-Visible Spectrophotometer). The quantity of siderophores in cell-free supernatant was measured as percent siderophores units (psu), using the following formula (1) [72,85]:

(1) $$\mathrm{psu}=\frac{\left(A_{r}-A_{s}\right)}{A_{r}}\times 100$$

where, *A_r_* is the absorbance of the reference and *A_s_* is the absorbance of the sample (CAS solution and cell free supernatant of the bacterial culture).

CTS (catechol-type siderophores) levels in bacterial supernatants were determined based on the Arnow assay [23], and HTS (hydroxamate -type siderophores) levels were evaluated as described by Csáky [24], with 2,3-dihydroxybenzoic acid and hydroxylamine hydrochloride as standards, respectively.

#### 4.3.3. Phosphate-Solubilization

Phosphate-solubilization was determined qualitatively by plating bacteria on Pikovskaya agar [86] containing precipitated tricalcium phosphate Ca_3_(PO_4_)_2_. The results were expressed as a solubilization index (SI), which was estimated based on the ratio of the halo diameter (HD) to the colony diameter (CD) (2) [87,88]:

(2) $$SI=\frac{CD+HD}{CD}\times 100$$

The isolates were grouped as low (*SI* < 2), middle (2 < *SI* ≤ 4), and high solubilizers (*SI* > 4), according to Berraquero et al. [25].

#### 4.3.4. Oligonitrotrophic and Nitrogen-Fixation Screening

For the screening of oligonitrotrophic isolates, the fresh colony was inoculated onto nitrogen-free Ashby’s mannitol agar [89] and Nf agar (Nitrogen-free agar) [90], respectively. The isolate showed growth in Ashby’s mannitol agar and Nf agar made from green to blue was presumed as positive isolate. Then, the positive strain was picked for the second-generation test—amplification of the *nifH* gene [91]. Genomic DNA of each bacterial strain was extracted using a MasterPure™ Complete DNA and RNA Purification Kit (MP Biomedicals, OH, USA), according to the manufacturer’s protocol. Amplification of the *nifH* gene was conducted with primers 19F (5′-GCIWTYTAYGGIAARGGIGG-3′) and 407R (5′-AAICCRCCRCAIACIACRTC-3′) [91]. The 20 µL reaction mixture consisted of 12.5 µL of 2× DreamTaq Green PCR Master Mix (Thermo Scientific), 1 µL of each primer (0.4 µM), 1 µL DNA (100 ng) and sterile MilliQ water. Polymerase chain reaction (PCR) was performed in the thermocycler (Professional 96 Basic Gradient. Biometra. Germany). The PCR conditions with 100 ng of template DNA were: 3 min at 95 °C, 30 s at 95 °C, 30 s at 50 °C, 1 min at 72 °C and 10 min at 72 °C for 30 cycles. Sterile milliQ water was used as a negative control and strain *Azotobacter vinelandii* DSMZ 2289 from the German collection (Deutsche Sammlung von Mikroorganismen und Zellkulturen GmbH) as a positive control. The amplified *nifH* gene was separated by 2% agarose gel electrophoresis and visualized on a UV transilluminator using the gel documentation system “Bio-Profil software” (Vilber Lourmat, France). In addition, the content of total nitrogen in the examined bacterial cultures (Nfb medium) after 24, 48 and 72 h of incubation at 28 °C was analyzed by the Kjeldahl method using the Analitik Jena Multi N/C 2100 analyzer. The nitrogen fixing efficiency was determined as the mg of nitrogen produced per gram of carbon utilized. The utilization of carbon was evaluated by burning in a stream of pure oxygen at a high temperature with a chemiluminescence detector [92,93,94].

### 4.4. Statistical Analysis

All experiments were performed in triplicate, except for Biolog GEN III assays. All relative values were presented as means ± standard deviation (SD). Datasets were subjected to statistical analysis using STATISTICA.PL (13.1) software (StatSoft Inc., Tulsa, OK, USA). The data from Biolog GEN III experiments were combined in a single matrix, being represented as a positive integer, OmniLog TM units (OL units). To illustrate the Biolog results and metabolic profiles, the similarity patterns of sugar and amino acid utilization and chemical sensitivity assays between all the strains were presented based on individual heatmap graphs. The AWCD of all the strains were calculated, where AWCD was the sum of the differences between the OL units of the blank well (water) and substrate wells divided by 95 (the number of substrate wells in the GEN III microplates), after 120 h of incubation. For the substrates, Shannon diversity (H′) and Shannon evenness (E) indices were also assessed. Correlations between the determined parameters were assessed by one-tailed correlation analysis (Spearman’s rank correlation with Bonferroni correction), using mean data values. Moreover, a multivariate statistical method using PCA was performed to summarize the variability of the tested strains, and to determine the association among the measured activities. For Spearman’s rank correlation, PCA and heatmap analysis, all the data were standardized so that each score contributed equally to the analysis.

## 5. Conclusions

The negative effects of the use of fertilizers and pesticides for improving plant productivity are an important global problem. Such agricultural practices can cause damage to the environment, biodiversity and human and animal health. Therefore, the current study focused on the screening and biochemical characterization of bacteria inhabiting the endosphere of important plants. Phosphate solubilization, the production of IAA-like compounds and siderophores (CTS and HTS), as well as nitrogen fixation were observed, and the phenotypic profiles of strains were determined. *Delftia* sp. strain ZS2, *Delftia* sp. strain ZS6 and *Stenotrophomonas* strain ES2 showed promise to promote the plant growth. The tested strains were isolated from various, often very distant, plant species. The current study indicated that plants representing different species may be the host for the same genus of bacteria. Moreover, the PCA revealed a clear clustering of tested strains on the basis of their taxonomy in a relation to PGP features and properties. An important aspect of the current research is to search endophytic bacteria that could have a broad host range (e.g., *Delftia* sp.). Future research will be focused on assessing the synergistic effect of plant co-inoculation with bacteria isolated from the host and non-host plants. The combination of phenotypic and genetic analysis with metabolic tests will allow the most appropriate strains to be selected as effective components of biopreparations. The strains described in this study are promising candidates for the design of active microbial consortia promoting plant growth. In the future, selected strains and their mixtures will be further evaluated in greenhouse and field experiments, in natural soil and climate conditions.

## Figures and Tables

**Table 1:** Representative data of identification based on Biolog GEN III plates for 23 tested strains. A comparison with an identification based on the sequencing of the 16S rRNA gene was presented. The table presents the results of average well color development (AWCD), Shannon evenness (E) and Shannon diversity index (H′) values based on substrates used in Biolog GEN III. The values are means ± standard deviation (SD; *n* = 2).

Isolated from			Identification and Functional Diversity Indices							
Phylum		Species	Strain	GenBank Accession no.	16S rRNA	Biolog GEN III				
					Closest known Relative	Identification Based on Biolog GEN III	Similarity	AWCD	Shannon Diversity (H′)	Shannon Evenness (E)
***Monilophyta***		*Equisetum arvense*	ER1	KY486814	*Comamonas koreensis*	*Bordetella trematum*	0.68 ± 0.03	92.58 ± 0.81	3.93 ± 0.01	0.92 ± 0.00
			ES1	KY486815	*Rhizobium* sp.	*Rhizobium radiobacter*	0.54 ± 0.03	113.88 ± 0.07	4.30 ± 0.00	0.97 ± 0.00
			ES2	KY486848	*Stenotrophomonas maltophilia*	*Stenotrophomonas maltophilia*	0.65 ± 0.07	139.85 ± 0.30	4.08 ± 0.00	0.93 ± 0.00
			ES4	KY486828	*Brevundimonas* sp.	*Brevundimonas vesicularis*	0.58 ± 0.03	77.60 ± 0.51	3.75 ± 0.01	0.90 ± 0.01
			ES7	KY486816	*Brevundimonas* sp.	*Brevundimonas vesicularis*	0.56 ± 0.04	81.86 ± 0.22	3.88 ± 0.00	0.90 ± 0.00
***Spermatophyta***	**Monocots**	*Zea mays*	ZR1	KY486807	*Novosphingobium resinovorum*	*Rhizobium radiobacter*	0.53 ± 0.00	132.17 ± 0.05	4.34 ± 0.00	0.98 ± 0.00
			ZR3	KY486832	*Delftia acidovorans*	*Delftia acidovorans*	0.57 ± 0.03	130.32 ± 1.00	4.18 ± 0.01	0.92 ± 0.00
			ZR4	KY486833	*Delftia acidovorans*	*Delftia acidovorans*	0.71 ± 0.02	130.27 ± 0.11	4.11 ± 0.00	0.93 ± 0.00
			ZR5	KY486808	*Stenotrophomonas* sp.	*Stenotrophomonas maltophilia*	0.66 ± 0.05	126.79 ± 0.95	4.09 ± 0.01	0.94 ± 0.00
			ZS2	KY486834	*Delftia acidovorans*	*Delftia acidovorans*	0.76 ± 0.04	120.73 ± 0.47	4.07 ± 0.00	0.92 ± 0.00
			ZS5	KY486835	*Delftia acidovorans*	*Delftia acidovorans*	0.54 ± 0.01	126.73 ± 0.12	4.12 ± 0.00	0.92 ± 0.00
			ZS6	KY486831	*Delftia* sp.	*Delftia acidovorans*	0.71 ± 0.02	115.90 ± 0.94	4.05 ± 0.00	0.93 ± 0.00
		*Secale cereale*	SR1	KY486822	*Delftia* sp.	*Delftia acidovorans*	0.65 ± 0.01	112.13 ± 0.14	3.97 ± 0.00	0.94 ± 0.00
			SR3	KY486810	*Delftia acidovorans*	*Delftia acidovorans*	0.65 ± 0.00	113.33 ± 0.54	3.98 ± 0.00	0.94 ± 0.00
			SS5	KY486813	*Delftia acidovorans*	*Delftia acidovorans*	0.67 ± 0.03	110.89 ± 0.25	3.96 ± 0.00	0.94 ± 0.00
		*Triticum aestivum*	TS1	KY486817	*Delftia acidovorans*	*Delftia acidovorans*	0.62 ± 0.05	101.44 ± 0.17	3.93 ± 0.00	0.94 ± 0.00
			TS4	KY486820	*Delftia acidovorans*	*Delftia acidovorans*	0.74 ± 0.02	120.32 ± 0.02	4.04 ± 0.00	0.95 ± 0.00
	**Eudicots**	*Arctium lappa*	AR2	KY486811	*Collimonas pratensis*	*Achromobacter denitrificans/ruhlandii*	0.72 ± 0.01	118.07 ± 0.47	3.97 ± 0.00	0.93 ± 0.00
			AR3	KY486824	*Achromobacter xylosoxidans*	*Achromobacter ruhlandii/denitrificans*	0.72 ± 0.01	118.10 ± 0.59	3.96 ± 0.00	0.93 ± 0.00
			AR4	KY486847	*Stenotrophomonas maltophilia*	*Stenotrophomonas maltophilia*	0.74 ± 0.02	126.72 ± 1.15	4.09 ± 0.01	0.94 ±0.00
		*Vicia faba*	VR2	KY486805	*Variovorax paradoxus*	*Acidovorax cattleyae*	0.57 ± 0.03	102.86 ± 0.87	4.05 ± 0.00	0.95 ± 0.00
			VS3	KY486825	*Rhizobium* sp.	*Acidovorax cattleyae*	0.57 ± 0.03	103.61 ± 0.65	4.07 ± 0.00	0.96 ± 0.00
			VS4	KY486829	*Delftia acidovorans*	*Delftia acidovorans*	0.64 ± 0.02	119.92 ± 0.15	4.08 ± 0.00	0.92 ± 0.00

AWCD, average well color development; H′, Shannon diversity; E, Shannon evenness; the column “Similarity” indicates the degree of similarity physiological profile of the test strain with the strain deposited in the Biolog GEN III database. Strain number designation—Monilophyta: E, *Equisetum arvense* L. (horsetail); seed plants (Spermatophyta), monocotyledonous (Monocots): S, *Secale cereale* L. (rye); T, *Triticum aestivum* L. (wheat); Z, *Zea mays* L. (maize); seeds plants (Spermatophyta), dicotyledonous (Eudicots): A, *Arctium lappa* L. (burdock); V, *Vicia faba* L. (broad bean); part of plant: R, root; S, stem. The letter abbreviations of strain names are used throughout the publication.

**Table 2:** Features associated with potential plant growth promoting (PGP) activities of bacterial isolates: indole-3-acetic acid (IAA)-like compounds production, siderophores production and phosphate solubilization. The values are means ± standard deviation (SD; *n* = 3).

Strain		IAA-like Compounds Production (µg/mL)		Siderophores Production				SI
		Trp+	Trp-	Sid-CAS	Sid-CAS (psu)	HTS	CTS	
ER1	*C. koreensis*	8.72 ± 0.22	1.69 ± 0.04	+++	72.52 ± 0.22	1.45 ± 0.41	0.58 ± 0.09	0.00
ES1	*Rhizobium* sp.	0.75 ± 0.03	0.67 ± 0.04	+	34.57 ± 0.22	2.02 ± 0.07	0.01 ± 0.00	0.00
ES2	*S. maltophilia*	19.18 ± 0.20	4.26 ± 0.15	-	6.09 ± 0.08	0.38 ± 0.047	0.00	0.00
ES4	*Brevundimonas* sp.	6.51 ± 0.10	0.83 ± 0.03	+	25.10 ± 0.06	0.68 ± 0.01	0.00	0.00
ES7	*Brevundimonas* sp.	1.70 ± 0.03	0.35 ± 0.04	++	55.70 ± 0.08	0.38 ± 0.03	1.93 ± 0.03	0.00
ZR1	*N. resinovorum*	0.22 ± 0.03	0.17 ± 0.01	+	23.69 ± 0.10	0.33 ± 0.51	0.12 ± 0.01	2.82 ± 0.11
ZR3	*D. acidovorans*	2.65 ± 0.13	0.94 ± 0.07	−	00.00	00.00	00.00	0.00
ZR4	*D. acidovorans*	1.73 ± 0.17	0.73 ± 0.03	++	60.26 ± 0.15	2.04 ± 0.17	0.19 ± 0.02	0.00
ZR5	*Stenotrophomonas* sp.	8.27 ± 0.15	0.78 ± 0.02	−	0.00	0.00	0.00	0.00
ZS2	*D. acidovorans*	10.43 ± 0.31	0.77 ± 0.04	+++	81.62 ± 0.23	0.51 ± 0.20	0.49 ± 0.05	0.00
ZS5	*D. acidovorans*	5.58 ± 0.17	0.92 ± 0.02	+++	77.92 ± 0.07	1.02 ± 0.32	0.28 ± 0.02	0.00
ZS6	*Delftia* sp.	2.66 ± 0.27	1.07 ± 0.05	-	36.84 ± 0.13	0.21 ± 0.04	0.00	4.1 ± 0.11
SR1	*Delftia* sp.	14.53 ± 0.31	1.39 ± 0.05	++	69.87 ± 0.10	0.000	0.11 ± 0.01	0.00
SR3	*D. acidovorans*	2.41 ± 0.08	1.27 ± 0.03	++	44.74 ± 0.16	0.23 ± 0.06	1.00 ± 0.04	0.00
SS5	*D. acidovorans*	5.87 ± 0.09	0.95 ± 0.04	++	43.31 ± 0.19	1.24 ± 0.11	0.27 ± 0.06	0.00
TS1	*D. acidovorans*	9.62 ± 0.14	0.94 ± 0.02	−	0.00	0.00	0.00	0.00
TS4	*D. acidovorans*	22.51 ± 0.48	1.23 ± 0.02	−	0.00	0.00	0.00	0.00
AR2	*C. pratensis*	2.68 ± 0.08	0.46 ± 0.02	−	3.45 ± 0.20	0.26 ± 0.06	0.00	0.00
AR3	*A. xylosoxidans*	0.13 ± 0.01	0.00	++	40.31 ± 0.20	2.11 ± 0.40	0.00	0.00
AR4	*S. maltophilia*	20.60 ± 0.37	4.35 ± 0.08	−	0.00	0.00	0.00	0.00
VR2	*V. paradoxus*	1.26 ± 0.07	0.44 ± 0.03	++	59.64 ± 0.21	0.00	0.19 ± 0.03	0.00
VS3	*Rhizobium* sp.	0.56 ± 0.01	0.75 ± 0.03	−	0.00	0.00	0.00	2.18 ± 0.06
VS4	*D. acidovorans*	1.02 ± 0.76	0.76 ± 0.03	+++	71.83 ± 0.15	0.00	0.11 ± 0.01	0.00

Trp+, indole-3-acetic acid (IAA)-like compounds production in the presence of L-tryptophan; Trp-, IAA-like compounds production in the absence of L-tryptophan; Sid-CAS, qualitative production of siderophores (Sid) on agar with chrome azurol S (CAS), orange zone size: +++ large zone, ++ medium zone, + small zone, - no zone; Sid-CAS, quantitative production of siderophores production expressed as percent siderophores units (psu); HTS, quantitative production of hydroxamate-type siderophores (HTS); CTS, quantitative production of catechol-type siderophores (CTS); SI, phosphate solubilization index; Efficiency N/C: efficiency of nitrogen fix as the amount mg of nitrogen produced per gram of carbon utilized.

**Table 3:** Results of analysis regarding nitrogen fixing by tested bacteria (growth on Nfa and Ashby medium, presence of the *nifH* gene and efficiency of nitrogen fixing using the Kjeldahl method).

Strain No.	Growth		*nifH* Gene	N Concentrations and C Utilization; Efficiency Factor Obtained by N/C								
				24 h			48 h			72 h		
	Nfa	Ashby agar		Measured N	C utilized	Efficiency N/C	Measured N	C utilized	Efficiency N/C	Measured N	C utilized	Efficieny N/C
ER1	+++	++	+	1.84 ± 0.15	0.13 ± 0.01	14.15	2.85 ± 0.05	0.15 ± 0.01	19.00	3.44 ± 0.23	0.15 ± 0.03	22.93
ES1	+++	+++	+	2.51 ± 0.15	0.14 ± 0.01	17.93	3.65 ± 0.08	0.15 ± 0	24.33	4.80 ± 0.21	0.16 ± 0	30.00
ES2	+++	++	+	2.74 ± 0.15	0.15 ± 0.01	18.27	4.14 ± 0.13	0.16 ± 0	25.88	4.34 ± 0.07	0.16 ± 0	27.13
ES4	++	+	+	2.28 ± 0.07	0.15 ± 0.01	15.20	2.61 ± 0.19	0.15 ± 0.01	17.40	3.81 ± 0.16	0.16 ± 0.01	23.81
ES7	++	+	+	2.26 ± 0.08	0.14 ± 0.02	16.14	3.23 ± 0.10	0.15 ± 0.02	21.53	4.01 ± 0.19	0.16 ± 0.01	25.06
ZR1	+++	+++	+	3.53 ± 0.14	0.12 ± 0.02	29.42	4.26 ± 0.03	0.13 ± 0.02	32.77	5.16 ± 0.17	0.15 ± 0	34.40
ZR3	++	++	+	1.93 ± 0.08	0.12 ± 0.02	16.08	2.63 ± 0.25	0.14 ± 0.03	18.79	3.28 ± 0.21	0.15 ± 0.02	21.87
ZR4	+	+	+	0.00	0.00	0.00	1.37 ± 0.14	0.14 ± 0.01	9.79	1.85 ± 0.07	0.16 ± 0.01	11.56
ZR5	+++	++	+	2.35 ± 0.09	0.13 ± 0.02	18.08	3.17 ± 0.08	0.13 ± 0.02	24.38	3.69 ± 0.33	0.13 ± 0.02	28.38
ZS2	+++	++	+	2.96 ± 0.09	0.14 ± 0.01	21.14	3.11 ± 0.01	0.14 ± 0	22.21	3.85 ± 0.15	0.14 ± 0	27.50
ZS5	++	++	+	2.61 ± 0.07	0.14 ± 0.01	19.64	2.88 ± 0.09	0.14 ± 0.01	20.57	3.39 ± 0.25	0.15 ± 0.01	22.60
ZS6	+++	++	+	2.53 ± 0.09	0.12 ± 0.01	21.08	3.27 ± 0.09	0.14 ± 0.01	23.36	4.01 ± 0.09	0.15 ± 0.01	26.73
SR1	+++	++	+	3.25 ± 0.15	0.15 ± 0.01	21.67	3.99 ± 0.15	0.16 ± 0.01	24.94	4.73 ± 0.15	0.17 ± 0	27.82
SR3	++	+	+	1.82 ± 0.07	0.14 ± 0.02	13.00	2.56 ± 0.07	0.15 ± 0.01	17.07	3.30 ± 0.07	0.16 ± 0.01	20.63
SS5	++	+	+	2.39 ± 0.04	0.13 ± 0.01	18.38	3.27 ± 0.24	0.15 ± 0.01	21.80	3.69 ± 0.38	0.16 ± 0.01	23.06
TS1	++	+	+	2.12 ± 0.07	0.13 ± 0.02	16.31	2.99 ± 0.2	0.14 ± 0.01	21.36	3.33 ± 0.21	0.14 ± 0.01	23.79
TS4	+++	+++	+	4.53 ± 0.13	0.13 ± 0.02	34.85	5.43 ± 0.12	0.14 ± 0.01	38.79	5.91 ± 0.07	0.15 ± 0.01	39.40
AR2	+	+	-	0.00	0.00	0.00	0.00	0.00	0.00	0.00	0.00	0.00
AR3	+++	+++	+	3.34 ± 0.09	0.13 ± 0.01	25.69	5.02 ± 0.08	0.15 ± 0.02	33.47	5.99 ± 0.08	0.16 ± 0	37.44
AR4	+++	++	+	3.94 ± 0.05	0.14 ± 0.01	28.14	4.30 ± 0.08	0.15 ± 0.01	28.66	4.86 ± 0.10	0.15 ± 0.01	32.40
VR2	+++	+++	+	3.22 ± 0.05	0.13 ± 0.02	24.77	3.81 ± 0.05	0.14 ± 0	27.21	4.68 ± 0.05	0.15 ± 0	31.20
VS3	+++	+++	+	3.48± 0.14	0.13 ± 0.01	26.77	4.07 ± 0.14	0.13 ± 0.01	31.31	4.94 ± 0.014	0.15 ± 0	32.93
VS4	+++	+++	+	2.77 ± 0.06	0.13 ± 0.01	21.31	3.40 ± 0.06	0.13 ± 0.01	26.15	4.30 ± 0.06	0.15 ± 0	28.67

* Growth: +++ high growth; ++ medium growth; + low growth; – no growth; Kjeldahl method: Measured N—Measured nitrogen (mg/100 mL); C utilized—Carbon utilized (g/100 mL); Efficiency—amount in mg of N/g of carbon.

**Table 4:** Matrix correlation (Spearman correlation coefficients with the Bonferroni correction) between the different variables of the tested endophytic isolates.

Parameters	AWCD	E	H′	IAA Trp-	IAA Trp+	SI	psu	HTS	CTS	pH 6	pH 5	1% NaCl	4% NaCl	8% NaCl	Sugars	Amino Acids	Efficiency N/C
**AWCD**		0.11	0.80 *	0.05	0.07	0.07	−0.17	−0.13	−0.22	0.61 *	0.66 *	0.67 *	0.37	0.52 *	0.3	0.22	0.08
**E**	0.11		0.31	−0.11	−0.15	0.29	−0.46	−0.29	−0.26	−0.14	−0.30	−0.13	−0.36	−0.39	0.27	−0.13	0.48
**H′**	0.80 *	0.31		−0.07	−0.18	0.23	−0.10	−0.18	−0.07	0.14	0.30	0.18	−0.11	0.13	0.59	0.09	0.17
**IAA Trp-**	0.05	−0.11	−0.07		0.78 *	−0.16	−0.10	−0.24	−0.10	0.38	0.28	0.27	0.02	0.18	−0.14	−0.47	−0.18
**IAA Trp+**	0.07	−0.15	−0.18	0.78 *		−0.37	−0.13	−0.17	−0.18	0.44	0.36	0.39	0.33	0.36	−0.17	−0.37	−0.15
**SI**	0.07	0.29	0.23	−0.16	−0.37		−0.21	−0.20	−0.19	−0.40	−0.34	−0.34	−0.174	−0.45	0.16	−0.03	0.32
**psu**	−0.17	−0.46	−0.10	−0.10	−0.13	−0.21		0.39	0.81 *	−0.25	0.01	−0.25	−0.29	−0.12	−0.20	0.16	−0.17
**HTS**	−0.13	−0.29	−0.18	−0.24	−0.17	−0.20	0.39		0.29	−0.17	0.03	−0.14	0.19	0.01	−0.17	0.49	−0.31
**CTS**	−0.22	−0.26	−0.07	−0.1	−0.18	−0.19	0.81 *	0.29		−0.36	−0.22	−0.37	−0.45	−0.21	−0.02	0.10	−0.23
**pH 6**	0.61 *	−0.14	0.14	0.38	0.44	−0.40	−0.25	−0.17	−0.36		0.80 *	0.97 *	0.66 *	0.81 *	−0.19	0.05	−0.16
**pH 5**	0.66 *	−0.30	0.30	0.28	0.36	−0.34	0.01	0.03	−0.22	0.80 *		0.80 *	0.54	0.78 *	−0.22	0.04	−0.30
**1% NaCl**	0.67 *	−0.13	0.18	0.27	0.39	−0.34	−0.25	−0.14	−0.37	0.97 *	0.80 *		0.68 *	0.82 *	−0.224	0.13	−0.10
**4% NaCl**	0.37	−0.36	−0.11	0.02	0.33	−0.17	−0.29	0.19	−0.45	0.66 *	0.54	0.68 *		0.73 *	−0.14	0.14	−0.14
**8% NaCl**	0.52	−0.39	0.13	0.18	0.36	−0.45	−0.12	0.01	−0.21	0.81 *	0.78 *	0.82 *	0.73 *		−0.08	0.124	−0.18
**Sugars**	0.30	0.27	0.59	−0.14	−0.17	0.16	−0.20	−0.17	−0.02	−0.19	−0.22	−0.22	−0.14	−0.08		−0.07	0.29
**Amino acids**	0.22	−0.13	0.09	−0.47	−0.37	−0.03	0.16	0.49	0.10	0.05	0.04	0.13	0.14	0.12	−0.07		−0.10
**Efficiency N/C**	0.08	0.48	0.17	−0.18	−0.15	0.32	−0.17	−0.31	−0.23	−0.16	−0.30	−0.10	−0.14	−0.18	0.29	−0.10	

Significant correlations where *p* < 0.0029 are marked with an asterisk and are in bold. The data were standardized. AWCD, average well color development; H′, Shannon diversity; E, Shannon evenness; IAA-like compounds Trp+, indole-3-acetic acid production in the presence of L-tryptophan; f Trp-, indole-3-acetic acid production in the absence of L-tryptophan; SI, phosphate solubilization index; psu, percent siderophores units; HTS, quantitative production of hydroxamate-type siderophores; CTS, quantitative production of catechol-type siderophores.

## Supplementary Materials

Supplementary materials can be found at https://www.mdpi.com/1422-0067/20/21/5283/s1.

## Abbreviations

PGPE	Plant Growth-Promoting Endophytes
PGPB	Plant Growth-Promoting Bacteria
PGP activities	Plant Growth-Promoting activities
AWCD	Average well color development
H′	Shannon diversity
E	Shannon evenness
LB medium	Luria-Bertani medium
ACC	Aminocyclopropane-1-carboxylic acid
HCN	Hydrogen cyanide
ISR	Induced systemic resistance
Nfa	Nitrogen-free agar
BTB	Bromothymol blue
IAA	Indole-3-acetic acid
Trp	L-tryptophan
CAS	Chrome Azurol S
PSU	Percent siderophores unit
HTS	Hydroxamate-type siderophores
CTS	Catechol-type siderophores
SI	Phosphate solubilization index
PCA	Principal component analysis
ROS	Reactive oxygen species
SOS	Salt overly sensitive
ABA	Abscisic acid
