# Supplementary figures and images for: Endophytic Bacteria Potentially Promote Plant Growth by Synthesizing Different Metabolites and their Phenotypic/Physiological Profiles in the Biolog GEN III MicroPlateTM Test
Source: Int J Mol Sci. 2019 Oct 24;20(21):5283. doi: 10.3390/ijms20215283 (PMC6862297; doi:10.3390/ijms20215283)

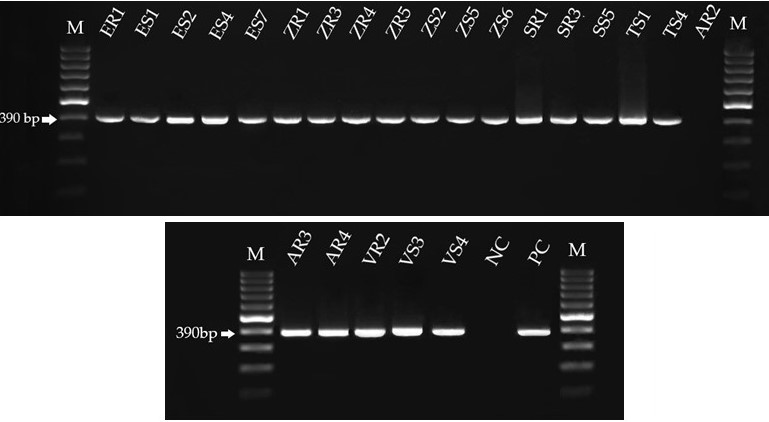

Supplement: Supplementary file 1 [file ijms-20-05283-s001.zip › ijms-625005-SI.jpg]
